# Covalent Immobilization of Polyaniline Doped with Ag^+^ or Cu^2+^ on Carbon Nanotubes for Ethylene Chemical Sensing

**DOI:** 10.3390/nano11081993

**Published:** 2021-08-03

**Authors:** Hagai Klein, Karthik Ananth Mani, Vinay Chauhan, Noga Yaakov, Franziska Grzegorzewski, Abraham J. Domb, Guy Mechrez

**Affiliations:** 1 Department of Food Sciences, Institute of Postharvest and Food Sciences, Agricultural Research Organization (ARO), Volcani Institute, 68 HaMaccabim Road, Rishon Lezion 7505101, Israel; Hagai.Klain@mail.huji.ac.il (H.K.); karthik.mani@mail.huji.ac.il (K.A.M.); chauhanvinay1985@gmail.com (V.C.); nogay@agri.gov.il (N.Y.); franziska@volcani.agri.gov.il (F.G.); 2 The School of Pharmacy, Faculty of Medicine the Hebrew, University of Jerusalem, Ein Karem, Jerusalem 9112102, Israel; avid@ekmd.huji.ac.il; 3 Institute of Biochemistry, Food Science and Nutrition, The Robert H. Smith Faculty of Agriculture, Food and Environment, The Hebrew University of Jerusalem, POB 12, Rehovot 7610001, Israel

**Keywords:** CNTs, polyaniline, ethylene, chemoresistors

## Abstract

Multi-walled carbon nanotubes (MWCNTs) are promising materials for chemical gas sensing because of their high electrical and mechanical properties and significant sensitivity to changes in the local environment. However, high-content MWCNT films suffer from the low tunability of the electrical resistance, which is crucial for high chemoresistive sensing performance. This study reports the conjugation of MWCNTs and oligomers of polyaniline (PANI) doped with Ag^+^ or Cu^2+^ incorporated into a PVC/polyacrylate. MWCNTs were sonicated in n-methyl pyrrolidine (NMP), and PANI was conjugated via a 1-ethyl-3-(3-dimethylaminopropyl) carbodiimide and an *N*-hydroxysuccinimide (EDC/NHS) process. MWCNT/PANI Ag^+^ or Cu^2+^ conjugates were doped to form a coordinate bond. The doped conjugates were successfully incorporated into the PVC/polyacrylate. These MWCNT/PANI conjugates doped were exposed to different concentrations of ethylene gas to examine their feasibility for ethylene detection.

## 1. Introduction

One of the main challenges to the preparation of polymer nanocomposite materials is the ability to obtain a homogeneous dispersion of the nano-filler in the polymer matrix [1,2,3]. Over the last few decades, carbon nanotubes (CNT’s) have attracted a great deal of attention due to their extraordinary mechanical and electrical properties. CNTs are an emerging class of materials for the chemical sensing of gases and volatile organic compounds [4,5,6,7,8,9,10]. Their very high aspect ratio makes them suitable for a wide range of applications. CNTs have the advantage of working at room temperature [11], and material costs are usually low. Although they have very high mechanical and electrical properties, it is highly challenging to capitalize on their advantages because of insufficient dispersion of the CNTs. This poor dispersion of CNTs leads to aggregation, which makes it harder to utilize them. Therefore, most CNT applications involve combining the CNTs with other materials such as polymers [12,13]. The incorporation of CNTs in a polymer matrix can produce a synergistic effect; i.e., their flexibility and low density, together with the mechanical and electrical properties of the CNTs lead to better dispersion of the CNTs, thus enhancing their applicability. Examples of applications of CNT/polymer nanocomposites include thin films [14] and conductive foam [15].

Chemoresistors rely on the direct chemical interaction between the sensing material and the analyte. The sensing material is deposited between two electrodes or coats a set of interdigitated electrodes connected to a potentiostat. Changes in the inherent electrical resistance of the electric circuit are measured as changes in the local chemical environment of the sensing material; i.e., interactions with the analyte lead to changes in the sensor’s resistance, which are proportional to the amount of analyte present. Thus, quantification of the analyte is feasible [16,17]. Given their extraordinary mechanical and electrical properties, multi-walled carbon nanotubes (MWCNTs) are an emerging class of materials for chemical sensing of gases and volatile organic compounds [4,5,6,7,8,9,10]. Their very high aspect ratio increases the adsorption rate of gases and leads to enhanced sensitivity at analyte concentrations in the ppm range and the rapid response time of the sensor.

Esser et al. presented a chemoresistive Cu^+^ complex based MWCNT sensor that can selectively detect sub-ppm concentrations of ethylene [18]. One drawback of using free-standing MWCNTs films is its structural stability [19]. Thus, mechanical abrasion of the MWCNT film in long-term applications cannot completely be excluded. This may result in non-linear responses and baseline drifts over the life of the sensor and causes a marked decline in sensor performance. Moreover, the relatively low intrinsic electrical resistance of MWCNTs produces a lower variation in resistivity upon adsorption of the analyte, which can end up in reduced sensitivity. Active materials are the most crucial factor governing the electrochemical performance of super-capacitors. Generally, there are three types of active materials: carbon materials [20,21] conducting polymers [22,23,24] and transition metal oxides [25,26]. Shaalan et al. synthesized defect-induced CNTs by the PECVD technique for ethylene detection at low operating temperatures and very low gas concentrations [27]. Vong et al. designed and developed the artificial metalloenzymes ethylene probe (AEP) to detect both exogenous and endogenous-induced changes to ethylene biosynthesis in fruit and leaves [28]. In another work, CeOx-SnO_2_ nanostructures were produced by a co-precipitation method, in which CeOx nano-crystallites containing Ce^3+^ and Ce^4+^ mixed oxidation states formed a composite structure with SnO_2_. These nanocomposites exhibited a high ethylene response, a short response time, a low detection range (0.3–10 ppm), and a high selectivity [29]. Fong et al. made a palladium (Pd) complex where the Pd cycling between Pd(0) and Pd(II) took place through Wacker oxidation and increased the electrical resistance [30]. Another work by Ishihara et al. used Wacker oxidation to sense ethylene. It utilized SWCNT with Wacker oxidation in two steps. First, ethylene was converted into acetaldehyde, which was followed by a reaction with hydroxylamine hydrochloride that emitted an HCl. The HCl reacted as a doping agent with SWCNT and decreased the electrical resistance [31]. Conducting polymers have the advantages of lower cost and high charge density in comparison to carbon materials and metal oxides. The synthesis and electrochemical properties of conducting polymers have been extensively studied and used. Among these various conducting polymers, polyaniline (PANI) has mostly been studied as a potential energy-storing material with unique doping/dedoping behavior, intrinsic electrical conductivity, facile synthesis, and environmental stability. Metal cations can convert into nitrogen atoms of the benzenoid group of PANI. Transition-metal ions with a relatively high electrode potential, such as Ag^+^ or Cu^2+^, are able to directly oxidize benzenoid groups of the polymer; i.e., induce the emeraldine base (EB) of PANI to pernigraniline (PB) conversion [32]. It should be noted that Ag^+^ or Cu^2+^ doped film possesses a conductivity of about 10^−4^ S/cm. It is well known that ethylene forms a number of reversible complexes with a large range of transition metals. High affinity has been reported for d^10^ transition metal ions, such as Cu^+^, Ag^+^, Cu^2+^, Ni^3+^, Pt^2+^, Rh^3+^, and Os^4+^ [33,34,35,36]. The binding can be generally described as a σ-donation/π backbonding of electron density between the olefin and the empty and filled metal d-orbitals, respectively [37]. These reversible interactions, which also exist in the ETR-1-ethylene complex and were implemented by Swager et al. for ethylene sensing [18,30], were the key parameters implemented to obtain specific ethylene-sensor interaction in this study.

This study presents a covalent immobilization of polyaniline doped with Ag^+^ or Cu^2+^ on carboxylated MWCNTs (MWCNTs-COOH) for ethylene chemical sensing. The carboxylated MWCNTs (0.06 wt%) are first dispersed in NMP by 5 min of sonication. Oligomers of polyaniline (PANI) are added to this solution and covalently immobilized to the MWCNTs with 1-ethyl-3-(3-dimethylaminopropyl) carbodiimide and *N*-hydroxysuccinimide (EDC/NHS). The conjugated MWCNT/PANI are doped with Ag^+^ or Cu^2+^ and convert the PANI from an emeraldine base (EB) into a pernigraniline base (PB) to achieve a specific interaction between the ethylene and the resistor based on the formation of reversible complexes between the ethylene and the doped MWCNT/PANI. After the immobilization of MWCNT/PANI, the resulting MWNNT/PANI conjugates are embedded into a PVC/polyacrylate by another 5 min of sonication. Since the MWCNT/PANI conjugate may be highly conductive due to the conductivity of both MWCNT and PANI, the incorporation of the MWCNT/PANI doped with Ag^+^ or Cu^2+^ in a PVC/polyacrylate matrix makes it possible to tune the electrical resistance of the system to meet the demands of ethylene detection. The combination of MWCNT/PANI conjugated with PVC/polyacrylate can form an electric network of MWCNT/PANI within the PVC/polyacrylate, thus making it applicable for chemical sensing. Below, we present the results of ethylene sensing through a MWCNT/PANI conjugate doped with Ag^+^ or Cu^2+^ embedded with PVC/polyacrylate. Different concentrations of ethylene change the electric resistance, thus confirming its feasibility for ethylene sensing.

## 2. Materials and Methods

### 2.1. Materials and Reagents

Aniline monomer (99%), ammonium persulfate (≥98%), ammonium hydroxide solution (28.0–30.0%), *N*-(3-Dimethylaminopropyl)-*N*′-ethylcarbodiimide hydrochloride (EDC), *N*-hydroxysuccinimide (NHS), silver nitrate (>99%), and copper (II) nitrate trihydrate (>99%) were purchased from Sigma-Aldrich (St. Louis, MO, USA) and used without further purification. 1-Methyl-2-pyrrolidinone (NMP) (99.5%) was purchased from Acros Organics (Geel, Belgium). The carboxyl-functionalized MWCNTs had an average diameter of 9.5 nm and an average length of 1.5 μm purchased from Sigma Aldrich (St. Louis, MO, USA). The PVC/polyacrylate was purchased from Rowa group (Pinneberg, Germany). Millipore water was used in the experiments.

### 2.2. Synthesis of Polyaniline (PANI) Oligomers

The aniline oligomers were synthesized according to Stejskal and Trchova [38] by oxidation of an aniline monomer (0.2 mol L^−1^) with ammonium persulfate (0.2 mol L^−1^) in 1 mol L^−1^ ammonium hydroxide at pH > 7 overnight. The reaction was carried out in 500 mL volumes. After completion of the reaction, the oligomers in the form of solids were isolated by filtration, rinsed with water, and dried overnight in a vacuum oven at 35 °C.

### 2.3. Preparation of MWCNT/PANI Conjugates

The synthesis of MWCNT/PANI conjugates took place in two steps. First, carboxyl-functionalized MWCNTs were reacted with polyaniline oligomers at room temperature in the presence of EDC/NHS as a coupling reagent and NMP as a solvent. The reaction was completed overnight and yielded the amide-linked PANI on the surface of MWCNTs. In the final step, the MWCNT/PANI conjugate was reacted with a metal ion solution AgNO_3_ or Cu(NO_3_)_2_ in a weight ratio 1:1 by stirring in NMP and kept overnight, which yielded a metal coordinated MWCNT/PANI conjugate. Five different MWCNT/PANI conjugates were made in ratios of MWCNT:PANI 10:1, 6:1, 3:1, 1:1, and 1:1.5, respectively.

### 2.4. UV-Vis Spectroscopy

The UV-visible spectra in PANI oligomers N-methylpyrrolidone were recorded with a Synergy™ Neo2 Multi-Mode (Winooski, VT, USA) Microplate Reader.

### 2.5. Fourier Transform Infrared Spectroscopy (FTIR)

The MWCNT, PANI, and its surface modification was verified using attenuated total reflectance Fourier transform infrared (ATR-FTIR) spectroscopy. Spectra were recorded on a Thermo 6700 FTIR instrument (Bruker, Billerica, MA, USA) equipped with a smart iTR diamond ATR device.

### 2.6. High-Resolution Scanning Electron Microscopy (HR-SEM)

Measurements were performed using a MIRA3 field-emission SEM microscope (Tescan, Brno, Czech Republic) with an acceleration voltage of 5.0 kV and an in-beam detector. Liquid samples were drop-cast on a conductive double stick carbon tape and dried in ambient conditions. Prior to imaging, a thin layer of gold–palladium was coated onto the samples to render them electrically conductive and to avoid surface charging by the electron beam. Energy-dispersive X-ray spectrometry (EDX) elemental analysis was performed on a MIRA3 SEM at 10 kV using an Oxford Instruments analyzer with AZtec software (Concord, MA, USA). STEM measurements were performed using a MIRA3 STEM with an acceleration voltage of 30.0 kV and a STEM bright detector.

### 2.7. Scanning Electron Cryomicroscopy (Cryo-SEM)

Scanning electron cryomicroscopy (Cryo-SEM) analysis was performed on a JSM-7800F Schottky Field Emission Scanning Electron Microscope (Jeol Ltd., Tokyo, Japan). A small droplet of the freshly mixed solution was placed on the sample holder between two rivets, quickly frozen in liquid nitrogen for a few seconds, and transferred to the preparation chamber where it was fractured (at −140 °C). The revealed fractured surface was sublimed at −90 °C for 10 min to eliminate any presence of condensed ice and then coated with platinum. The temperature of the sample was kept constant at −140 °C. Images were acquired with secondary electrons (SE) at an accelerating voltage of 1 to 15 kV at a working distance of maximum of 10.1 mm.

### 2.8. Cryogenic Transmission Electron Microscopy (Cryo-TEM)

Samples were loaded onto grids that were blotted and plunged into liquid ethane using a Gatan CP3 automated plunger and stored in liquid nitrogen until use. Frozen specimens were transferred to a Gatan 914 cryo-holder and maintained at temperatures below −176 °C inside the microscope. Samples were inspected with a Tecnai G2 microscope (Thermo Fisher, Waltham, MA, USA) with an acceleration voltage of 120 kV, which is equipped with a cryobox decontaminator. Images were acquired using a digital micrograph (Gatan) at different resolutions.

### 2.9. Thermogravimetric (TGA) Analysis

TGA of the polymer films was measured with a PerkinElmer Pyris 1 TGA instrument (Waltham, MA, USA) under N_2_ (50 mL/min) at a heating rate of 10 °C/min before and after SNP grafting. Weight loss curves were recorded from room temperature to 800 °C for a single specimen.

### 2.10. Design of the Chemoresistor Sensing Lab (CSL)

The CSL is a micro lab (TestView Ltd. Migdal HaEmek, Israel), which was designed and dedicated specifically for the characterization of sensing behavior and sensor development. The system contains components that together create the right environmental conditions for sensing. Briefly, the Source Measure Unit (SMU) (a) measures the electrical resistance. The diluter (b) prepares the right concentration of ethylene and transfers it to the reservoir (c) through all the valves and pipes (d) to the chamber (e) where all the sensors are located.

#### 2.10.1. Source Measure Unit (SMU)

The chemoresistor detection mechanism is based on the ability to develop an electrical resistor that exhibits changes in its electrical resistance correlated to the amount of absorbed gas. The SMU measures the changes in the electrical resistance of any given resistor. The SMU is a small device that can force voltage or current and measure the complementary criteria respectively. In our case, we forced a fixed current (*I*) and measured the voltage (*V*) under this current. The equation $R=\frac{V}{I}$ was used to calculate the electrical resistance (*R*) of the target materials. For the chemoresistor, a response to a specific analyte was reflected in changes in the electrical resistance. When different concentrations of analyte produce different responses, i.e., different changes in the electrical resistance, the analyte can be sensed.

#### 2.10.2. Diluter and Mass-Flow Controller

Each test started with the diluter to prepare a specific concentration of ethylene. Each test examined different concentrations of ethylene to obtain a different response from different concentrations. To change the gas concentrations, the diluter can control the flow of the gases by a mass flow controller. Specifically, when gas enters through the inlet, it changes the temperature inside the controller, and these changes are translated into voltage changes ranging from 0.5 to 5 V. These voltage changes displace a diaphragm that controls the flow of the gases. In our tests, nitrogen had a flow of 10 L/min, whereas ethylene flowed through the diluter at a speed of 2–10 mL/min. To achieve greater control of the ethylene concentrations, different size cylinders were chosen. For high concentrations (ppm levels), a full ethylene cylinder was used, and for lower concentrations (ppb levels), a 100 ppm cylinder was used.

#### 2.10.3. The Reservoir

The diluter can control the concentrations of ethylene, but it has a very small capacity. To overcome this problem, we designed a reservoir with a capacity of 12 L. The diluter transferred the mixture of nitrogen/ethylene directly to the reservoir. To make sure that the mixture of nitrogen/ethylene was homogeneous, we replaced the whole volume of the reservoir seven times. After seven cycles of volume replacement, the valves were shut at which point the mixture in the reservoir was ready for transfer into the chamber.

#### 2.10.4. The Chamber

When the reservoir has been loaded with the appropriate nitrogen/ethylene mixture, it is transferred directly to the chamber. The ethylene sensors are positioned on two boards within the chamber. To decrease the surface of the chamber and to make it more directly accessible to the gases, 8 different cylinders are placed within the chamber. During testing, the chamber goes through vacuum cycling to evacuate any molecules inside the chamber and then five minutes of exposure to the gases for the adsorption of the analyte (ethylene) for ethylene sensing.

#### 2.10.5. The Sensing System

The sensing devices were fabricated by applying porous conductive thin films on an interdigital transducer (IDT). The IDT was fabricated by standard photolithography on top of a silicon wafer on a SiO_2_ upper layer. Each board was designed to have 15 sensing stations. In our two board system, this allows for a total of 30 samples inside the chamber. Each chip has a drain and a source. The resistors are applied on top of the electrodes, and when the analyte is adsorbed, the electrical resistance changes, thus yielding an output response. Different concentrations of the analyte result in different changes in electrical resistance, which constitute the analyte sensing.

## 3. Results and Discussion

### 3.1. Synthesis of MWCNT/PANI Conjugates and Fabrication of the Sensing Device

**Figure 1:**
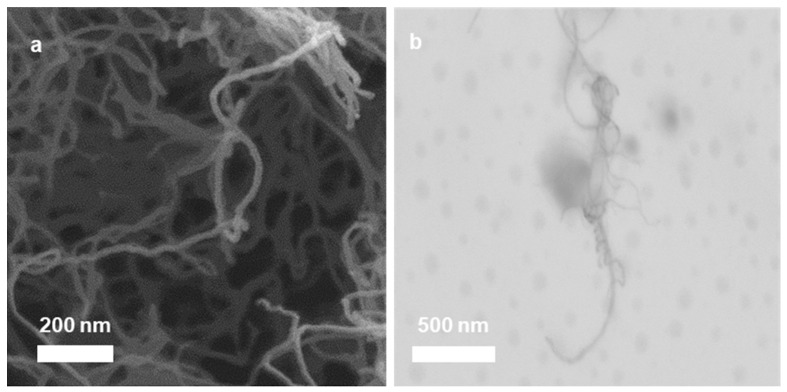
HRSEM micrograph (**a**) and STEM micrograph (**b**) of the carboxylated MWCNT, which were used in the current study.

Figure 1 depicts the typical structure of the carboxylated MWCNTs that were utilized in the current study. The carboxylated MWCNTs had an average diameter of 9.5 nm and an average length of 1.5 μm, giving them a relatively high aspect ratio of approximately 158. Furthermore, the surface area of the MWCNT was 220 m^2^/g, making it a strong candidate for gas sensing. The electrical resistance of the carboxylated MWCNTs was 7.0 ± 0.5 Ohm, which is sufficient for gas chemoresistive sensing.

**Figure 2:**
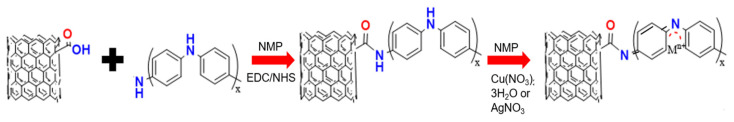
Chemical depiction of the conjugation and doping of MWCNT and PANI.

A schematic illustration of the synthesis procedure is presented in Figure 2. MWCNTs-COOH was reacted with PANI oligomers to form a conjugate of MWCNT/PANI. This reaction was carried out in NMP, which enables both individual dispersion of the MWCNTs-COOH and good dissolution of PANI. In step 1, the MWCNTs-COOH were dispersed in NMP via ultrasonication to obtain the individual dispersion of MWCNT in the NMP. The PANI oligomers were synthesized according to the procedure in Stejskal and Trchova (see Method) and were dissolved in NMP. Then, the PANI were covalently immobilized to the MWCNTs via EDC/NHS crosslinking in NMP. Different PANI/MWCNT ratios were studied. The conjugation reaction lasted overnight. The day after, the reaction mixture was filtrated with NMP solvent at 0.2 μm paper under vacuum. The obtained solid MWCNT-PANI oligomers conjugate was separated and used for further experiments. The filtrate was dispersed in NMP, and Ag^+^ or Cu^2+^ were doped inside the conjugate for 3 h. Since MWCNT had high conductivity, it could potentially create a conductive system for future applications. Since MWCNTs may be too conductive, a conjugation with PANI may help control the conductivity of the MWCNTs. PANI in an emeraldine base (EB) has lower conductivity than MWCNT and thus can control conductivity if covalently bonded to MWCNT. In addition, according to Dimitriev [39], doping with transitional metals such as Cu^2+^ can convert the PANI from EB to a pernigraniline base (PB), which has a different conductivity than EB, thus providing another potential route to control the new system.

### 3.2. UV-Vis Spectroscopy

**Figure 3:**
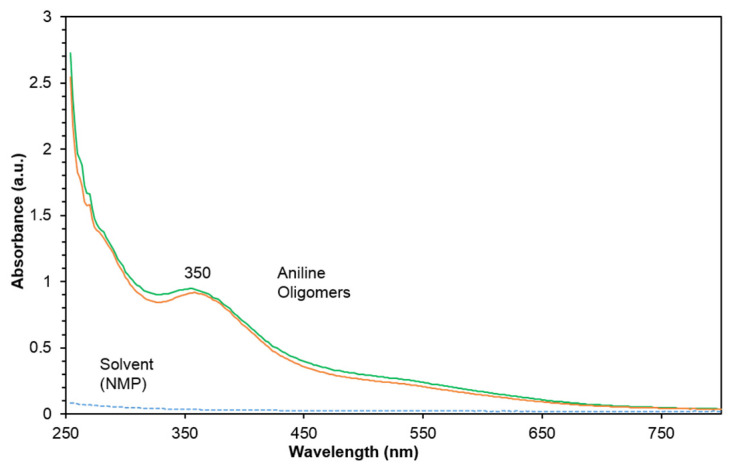
UV-vis spectrum of two batches of PANI (green and orange) and NMP (broken line).

The PANI oligomers were characterized by a UV-vis spectrophotometer. We synthesized two different batches of PANI (Figure 3 green and orange lines) and tested them in UV with an NMP solvent (broken line) as a control. The results showed maximum absorption around 350 nm with no other peak around 600 nm in both PANI batches and no absorption peak for the NMP solvent. According to Stejskal and Trchova, the PANI oligomers in the UV visible spectrum display a peak at a wavelength of around 348 nm, unlike PANI polymers that display a peak around 600 nm. These results may suggest the presence of aniline oligomers in the solution rather than long chains of polyaniline. Since UV-vis can only determine a range of PANI sizes, it is not appropriate for detecting the exact size and length of PANI chains. Further research should focus on detecting the exact size of PANI chains.

### 3.3. Fourier-Transform Infrared Spectroscopy (FTIR) Characterization

**Figure 4:**
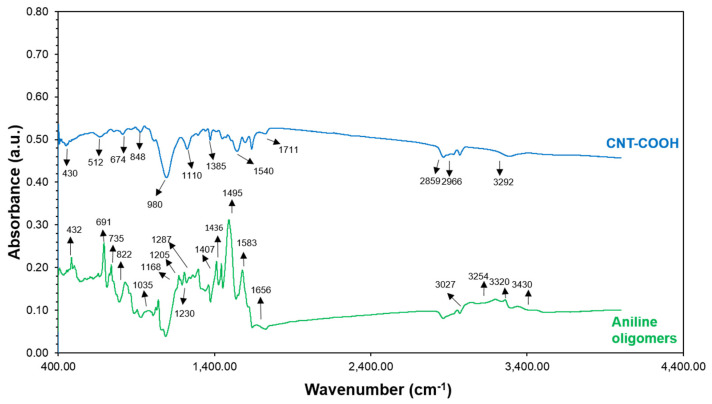
FTIR of carboxylated MWCNT (blue) and aniline oligomers (green).

**Figure 5:**
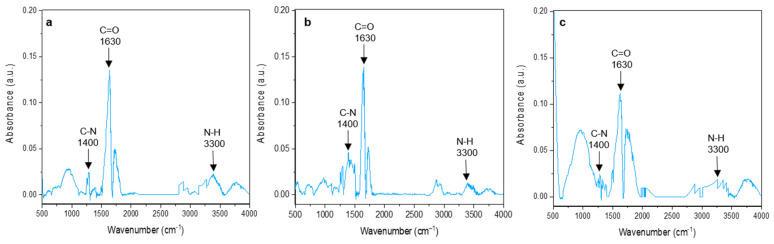
FTIR of three different conjugates of MWCNT/PANI at ratios of 3:1 (**a**); 6:1 (**b**); 10:1 (**c**).

The functional groups of the MWCNT/PANI conjugates, PANI oligomers, and MWCNT were characterized by ATR-FTIR spectroscopy. The results are summarized in Figure 4 and Figure 5. Peaks at 822, 735, and 691 cm^−1^ in the aniline oligomer’s spectrum (Figure 4) indicated C-H *out-of-plane* bending and *out-of-plane* ring deformations for the mono-substituted phenylene ring, respectively. A peak at 1436 cm^−1^ is responsible for the C=C stretching vibration of the substituted aromatic ring, which is characteristic to aniline oligomers. The peaks at 1583 and 1495 cm^−1^ can be attributed to quinonoid and benzenoid ring-stretching vibrations. For short chain aniline polymers, a peak for N–H scissoring indicates vibrations of aromatic amines or the presence of phenazine units, which was observed at 1656 cm^−1^. Peaks at 3440/3324 cm^−1^ and 3266/3195 cm^−1^ designate the asymmetric and symmetric free N–H stretching vibrations and hydrogen-bonded N–H vibrations, respectively. In the spectra of MWCNT (Figure 4), the peaks observed at 1711 and 1540 cm^−1^ were from C–O bands characteristic of carboxyl functional groups on the MWCNT-COOH surface. An FTIR spectra test was conducted to confirm the amide bond to prove the conjugation of the carboxylated MWCNT and PANI. The peaks proving the amide bond were at 1400 cm^−1^, 1640 cm^−1^, and 3300 cm^−1^ (Figure 5), which indicated a C–N, C=O, and N-H bond, respectively. In three different conjugates, the FTIR evidenced three different bands indicating an amide bond. An amide bond can only form by the bonding of carboxylic groups from MWCNTs and a secondary amine from PANI. These three peaks at 1400, 1600, and 3300 cm^−1^ in three different conjugates at different ratios may suggest that the conjugate process was successful and the PANI oligomers were conjugated with the carboxylated MWCNTs.

### 3.4. High-Resolution Scanning Electron Microscopy (HRSEM) and Energy-Dispersive X-ray Spectroscopy (EDX) Characterization

The resulting MWCNT/PANI were doped with Ag^+^ and Cu^2+^. A high affinity has been reported for d^10^ transition metal ions such as Cu^+^, Ag^+^, Cu^2+^, Ni^3+^, Pt^2+^, Rh^3+^, and Os^4+^ [33,34,35,36]. The binding can generally be described as a σ-donation/π backbonding of electron density between the olefin and the empty and filled metal d-orbitals, respectively [37]. These reversible interactions which also exist in the ETR-1-ethylene complex, and were implemented by Swager et al. for ethylene sensing [18,30], were the key parameters to obtaining the specific ethylene-sensor interactions in this study.

**Figure 6:**
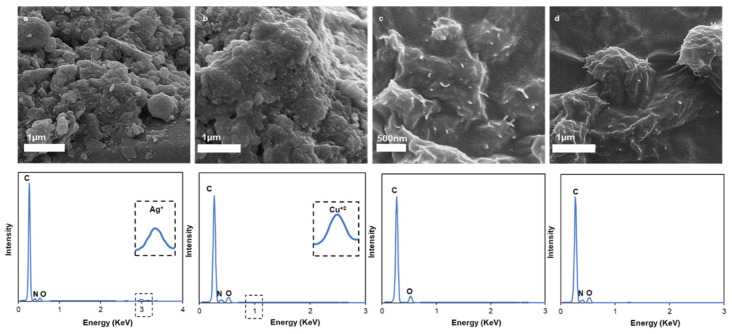
HRSEM images and EDX spectra of MWCNT/PANI/Ag^+^ (**a**); MWCNT/PANI/Cu^2+^ (**b**); MWCNTs (**c**); MWCNT/PANI conjugate (**d**).

The structural properties of the different MWCNT/PANI/Ag^+^ and MWCNT/PANI/Cu^2+^ conjugates were subjected to HRSEM. Figure 6 depicts the HRSEM images of MWCNT/PANI/Ag^+^, MWCNT/PANI/Cu^2+^, MWCNT only, and the MWCNT/PANI conjugate.

The structural properties of the different MWCNT conjugates can clearly be seen in the HRSEM micrographs (Figure 6a,b), although there was not a significant difference between the MWCNT (Figure 6c) and the MWCNT/PANI conjugate (Figure 6d). This may be explained by the lack of sensitivity of the samples themselves, since they were evaporated before the HRSEM analysis. This evaporation can make the solids aggregate more easily, thus preventing clearer differences from emerging. The MWCNT/PANI/Ag^+^ and MWCNT/PANI/Cu^2+^ conjugates had a very thick structure. These structures may be due to a sedimentation of Ag^+^ and Cu^2+^ salts in the solution. An EDX test was conducted to verify the presence of all the elementals, including carbon and oxygen from the MWCNTs, nitrogen from PANI, and Ag^+^ and Cu^2+^ from the doped system. Each sample exhibited a different elemental composition. The MWCNT were only indicated by peaks of C and O, which may indicate the presence of carboxylic groups from the MWCNTs. In the MWCNT/PANI conjugate, there was also a peak of N, which may suggest the presence of PANI together with MWCNTs. This is further confirmation of the successful conjugation of MWCNT/PANI. In addition, peaks of Ag^+^ and Cu^2+^ emerged, indicating the successful coordination bonding between PANI and the metals.

### 3.5. Scanning Electron Cryomicroscopy (Cryo-SEM) Characterization

**Figure 7:**
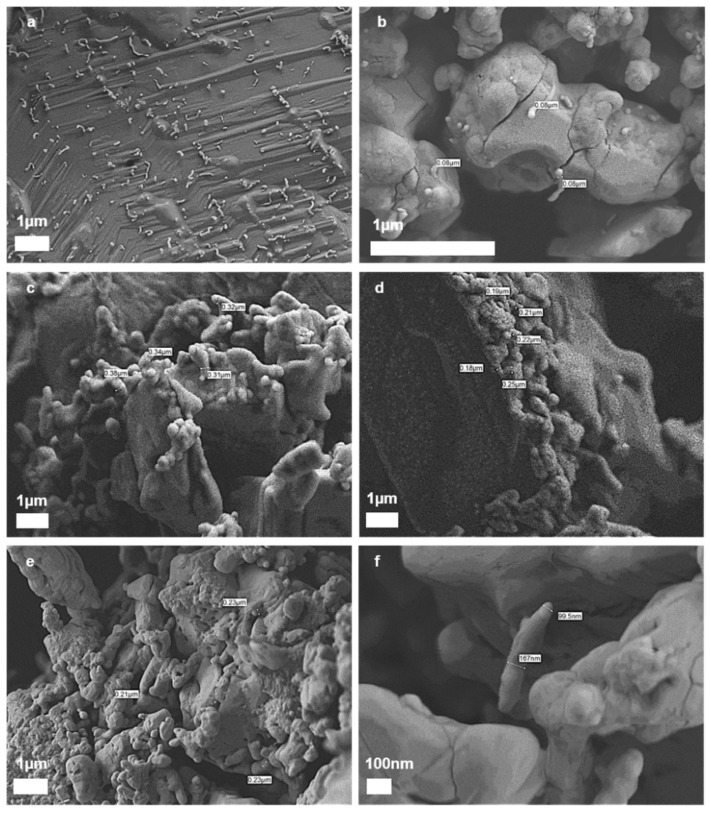
Cryo-SEM of MWCNT (**a**,**b**); MWCNT/PANI conjugates (**c**,**d**); MWCNT/PANI conjugates doped with Ag^+^ or Cu^2+^ (**e**,**f**).

The structure of the MWCNT/PANI conjugate dispersion in NMP was characterized by Cryo-SEM. The advantage of Cryo-SEM over SEM is there is no evaporation process in Cryo-SEM, which can display the samples as is. The Cryo-SEM images clearly indicated that the MWCNTs were fully dispersed in the NMP (Figure 7a,b). Furthermore, when PANI was added to the MWCNT, it became thicker than MWCNT alone. Thus, these Cryo-SEM images point to the possibility of polymerization through the MWCNTs, which provides another perspective on the conjugation of MWCNT/PANI.

The MWCNT diameter was approximately 80 nm according to the Cryo-SEM microscopy (Figure 7b). The micrographs of MWCNT/PANI conjugates dispersed in NMP are shown in Figure 7c,d. The MWCNTs conjugates were significantly thicker than the carboxylated MWCNT (Figure 7a,b) with a diameter of approximately 300 nm. The increased diameter after conjugation is a clear indication of the presence of PANI on the surface of the MWCNT and the successful covalent immobilization of the PANI oligomers on the MWCNT surfaces. Figure 7e,f show the MWCNT/PANI conjugates doped with Ag^+^ or Cu^2+^. No significant difference was found between the conjugates and the doped conjugates, which may indicate there was no structural difference between the non-doped MWCNT/PANI conjugate and the doped MWCNT/PANI conjugate.

### 3.6. Cryogenic Transmission Electron Microscopy (Cryo-TEM) Characterization

**Figure 8:**
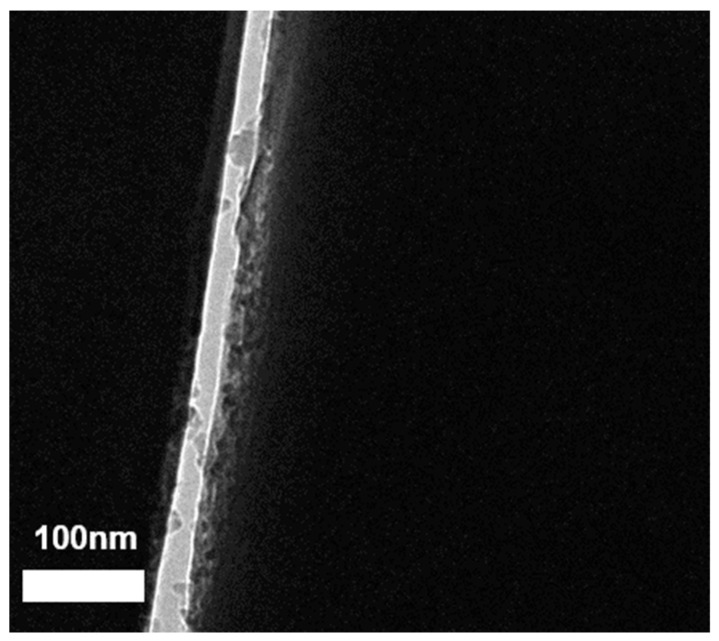
Cryo-TEM of single MWCNT/PANI/Ag^+^.

The structure of the MWCNT/PANI conjugates in dispersion was further characterized via Cryo-TEM (Figure 8). The Cryo-TEM microscopy serves to study the structure of the conjugates at extremely high resolution. Figure 8 depicts a Cryo-TEM image of doped PANI with Ag^+^ conjugated with MWCNT. Since the Cryo-TEM penetrates the samples, this provides another informative perspective. The Cryo-TEM image shows a single MWCNT with a “halo” around it. This is the signature of PANI, which coated the MWCNT, and it may also indicate the conjugated PANI immobilized to MWCNT.

The cryogenic microscopy characterization indicated that the PANI formed a thick layer on the surface of the MWCNT rather than the desired structure of polymer brushes conjugated to the surface of the MWCNTs.

### 3.7. Thermogravimetric (TGA) Analysis

TGA measures the glass temperature of the materials and constituted another confirmation of the successful immobilization of MWCNT/PANI.

**Figure 9:**
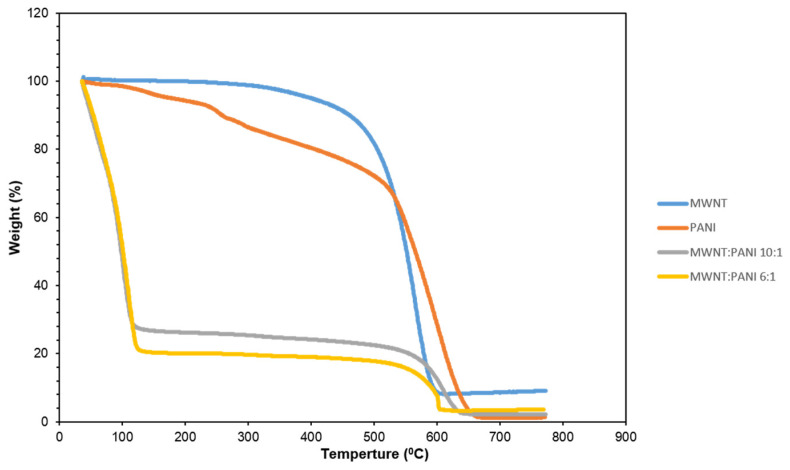
TGA results of MWCNT only (blue); PANI only (orange); MWCNT/PANI at a ratio of 10:1 (gray); MWCNT/PANI in ratio of 6:1 (yellow).

TGA (Figure 9) revealed a difference in weight (%) loss between MWCNT and PANI alone as compared to the MWCNT/PANI conjugate at different ratios of 10:1 and 6:1. This difference in weight loss may indicate different compounds because the same material should have the same weight loss profile. In addition, the TGA results showed that the conjugates were highly hygroscopic compared to MWCNT and PANI separately. This is an excellent example of how two different materials (i.e., MWCNT and PANI) can combine to form a new material with new properties (i.e., hygroscopic properties from the MWCNT/PANI conjugates). Combining the loss changes and hygroscopic changes in MWCNT and PANI separately, compared to the MWCNT/PANI conjugates, may also hint at a new material; i.e., a MWCNT/PANI conjugate.

### 3.8. MWCNT/PANI Conjugates Incorporated into PVC/Polyacrylate Matrix

The tuning of the electrical resistance of the system is crucial to meeting the demands of optimal ethylene detection of the sensor. To this end, we incorporated the MWCNT/PANI conjugates in a PVC/polyacrylate matrix. The electrical conductivity of the polymeric nanocomposites material can be accounted for by percolation theory [40,41,42]. At a given loading of the nanofillers in the polymer matrix, they form a continuous network within the polymer matrix. This concertation is termed the percolation threshold. Percolation makes it possible to tune the electrical resistance of the sensor and thus maximize the sensing parameters of the system in terms of detection limits.

**Figure 10:**
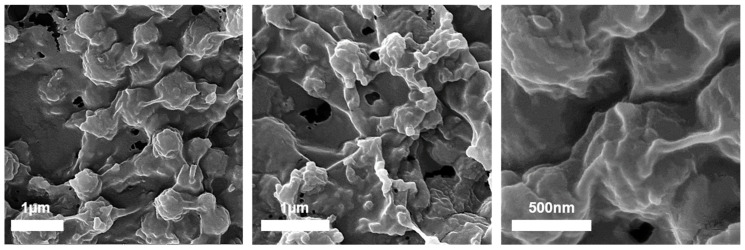
HRSEM images of MWCNT/PANI conjugates at a ratio of 1:1 doped with Ag^+^ embedded into PVC poly acrylate at a ratio of 1:10.

The MWCNT/PANI conjugates were incorporated into a PVC/polyacrylate matrix at ratios of 1:5, 1:10, 1:20, and 1:50. Figure 10 depicts HRSEM images of the MWCNT/PANI conjugates at a ratio of 1:1 embedded into PVC/polyacrylate at a ratio of 1:10. As shown in the HRSEM images, the conjugates were well dispersed into the polymeric matrix, since MWCNTs fully covered the polymeric matrix (Figure 10). The HRSEM images show a network of MWCNTs combined in PVC/polyacrylate, which may indicate a nanocomposite material with conductive material (MWCNT) incorporated into non-conductive material (PVC/polyacrylate). In addition, the HRSEM images showed aggregates of polymers together with the MWCNTs, making ‘ball like’ structures, which may have formed a barrier between the MWCNTs and interfered with the conductive network. Further research is needed to fully understand this phenomenon. The overall results of embedding the MWCNT/PANI conjugate into PVC/polyacrylate may imply that a conductive network of nano-composite material can be formed within the polymeric matrix, which could contribute to future conducive material applications.

### 3.9. MWCNT/PANI Conjugates in Ethylene Sensing

**Figure 11:**
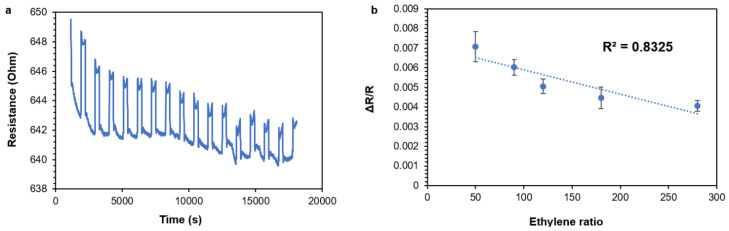
For MWCNT/PANI/Ag+ conjugates at a ratio of 1:1: The change in the electrical resistance (response) as a function of the ethylene concentration for low to high concentrations (**a**) and ΔR/R as a function of the ethylene (**b**).

Figure 11 depicts a sensing graph of ethylene. Five different concentrations of ethylene were used (50, 90, 120, 150, and 250 ppm) to evaluate the response. A response consisted of a change in the electric resistance. Changes in electric resistance as a function of changes in the ethylene concentration constituted the ethylene sensing.

As Figure 11 shows, there was an initial sensing of ethylene in the MWCNT/conjugate doped with Ag^+^ or Cu^2+^. Since five different concentrations of ethylene were measured, there was a change in the electric resistance (Figure 11a). In addition, there was a high correlation between the ethylene concentration changes and the electric resistance changes (Figure 11b). These initial sensing results may point to an applicable sensing system for future ethylene sensing. The mechanism of the sensing is based on a specific interaction between the MWCNT/conjugate doped with Ag^+^ or Cu^2+^ and the ethylene molecules. A high affinity has been reported for d^10^ transition metal ions, such as Cu^+^, Ag^+^, Cu^2+^, Ni^3+^, Pt^2+^, Rh^3+^, and Os^4+^ [28,29,30,31]. The binding can be generally described as a σ-donation/π backbonding of electron density between the olefin and the empty and filled metal d-orbitals, respectively [32].

The current limit of detection of the sensor was 50 ppm, and although this result is far from, being applicable at the industrial level, clear sensing behavior was observed. In future work, we are planning to present a MWCNT/PANI-based chemoresistor with a much lower detection limit. Again, this work only presents a feasibility analysis that can lead to the development of MWCNT/PANI-based sensors for ethylene detection. As can be seen in Figure 11, the response and the recovery time of the sensor was approximately 8 s on every exposure cycle, which is relativity fast and suitable for the demands inherent to the application. The sensors produced the same results as shown in Figure 11 after 14 months, thus demonstrating their long-term stability and robustness.

## 4. Conclusions

MWCNT/PANI conjugates doped with Ag^+^ and Cu^2+^ were fully characterized with UV-vis, FTIR, HRSEM, Cryo-SEM, Cryo-TEM, and TGA. The overall results suggest the successful dispersion of MWCNTs in NMP and a MWCNT/PANI conjugation. The findings also support the possibility of a coordination bond between PANI and the transitional metals Ag^+^ and Cu^2+^. The UV-vis test results suggested oligomer preparation, since the peak of absorption of the PANI was around 350 nm, which implies oligomer preparation. The FTIR test detected three peaks, which indicate an amide bond, which confirmed the conjugation between the MWCNTs and PANI. The HRSEM images showed fully dispersed NWNTs in NMP solvent and some aggregates that occurred after adding the MWCNT/PANI conjugates, which were doped with Ag^+^ or Cu^2+^. These aggregates are likely the result of salts, which may sink and form aggregates. Since the HRSEM images failed to show a significant difference between MWCNT alone and the MWCNT/PANI conjugate, and the Cryo-SEM images indicated a diameter difference between MWCNT alone and the MWCNT/PANI conjugate, this may indicate polymerization over the MWCNTs. The Cryo-TEM images support the feasibility of conjugation between MWCNTs and PANI. The TGA results demonstrated the different properties of the new conjugates. The MWCNT/PANI conjugates were incorporated successfully into the PVC/polyacrylate and could thus generate a conductive network within the polymeric matrix. Finally, the MWCNT/conjugate doped with Ag^+^ or Cu^2+^ successfully sensed ethylene gas, which may have potential applications for future ethylene sensing. Unlike other studies in the field of chemoresistive detection of ethylene, this work paves the way to tune the molecular weight of the “detection ligand” (i.e., the doped PANI) and confirms its ability to tune the electrical resistance of the sensing system by varying the MWCNT/PANI conjugate loading in a PVC/Polyacrylate matrix.

## Data Availability

Data are available upon request.
